# Evaluation of changes in the quality of extracted oil from olive fruits stored under different temperatures and time intervals

**DOI:** 10.1038/s41598-019-54088-z

**Published:** 2019-12-23

**Authors:** Samira Saffar Taluri, Seid Mahdi Jafari, Akbar Bahrami

**Affiliations:** 1 Department of Food Materials and Process Design Engineering, Gorgan University of Agricultural Sciences and Natural Resources, Gorgan, Iran; 2 Program of Applied science and Technology, Center for Excellence in Post-Harvest Technologies, North Carolina Agricultural and Technical State University, North Carolina Research Campus, Kannapolis, NC 28081 USA

**Keywords:** Biological techniques, Heat

## Abstract

Chilling and freezing injuries of olives harvested in geographically high elevated locations may affect the quality of olive, and subsequently lead to oil with a poor quality. This study was aiming to investigate the influence of whether changes and freezing condition on the quality of extracted olive oil. The olive Koroneiki cultivar obtained from two origins was stored at three different temperatures (20, 5 and −18 °C) before oil extraction and common analyses (oil yield, acidity, peroxide value, thiobarbitorik acid value, total phenolics level, and color) were carried out in different time intervals (0, 20, 40, and 60 days) in order to assess the olive oil quality. Our data revealed that longer storage times significantly (*P* < 0.05) decreased the quality of olive oil. The fruits remained at 20 °C provided the lowest oil quality in all parameters. For example, the acidity of olive oil at 20 °C was 177% higher than samples kept at 5 °C after 20 days of olive storage. The general trend for comparing the quality parameters of extracted oils from olives kept at different temperatures was −18 > 5 > 20 °C. No significant negative effect (*P* < 0.05) was found for the samples kept at −18 °C, compared to 5 °C. Also, the geographical source of olive had a statistically significant influence (*P* < 0.05) on the quality of olive oil.

## Introduction

Virgin olive oil is extracted from high quality and fresh olive fruits (*Olea europeae L*.) through mechanical processes and without use of preliminary refining, heating and solvents; it is considered as a valuable and nutritional vegetable oil^1,2^. That is among rare vegetable oils which can be used in its raw state directly as well. Virgin olive oil contains significant levels of nutritional compounds such as phenolic compounds, fatty acids, vitamins, and sterols^3^. Even extra virgin olive oil is claimed to have the highest quality among olive oils for its pleasing and especial flavor which is superior than other edible vegetable oils. Olive oil sensory uniqueness as well as its important nutritional properties have been the favorite of Mediterranean countries consumers and now throughout the world^4,5^.

Diverse varieties of olive fruits due to the variations in composition and overall properties have shown different behaviors in terms of quality loss when subjected to different temperatures. For example, Ruiz-Domínguez *et al*.^6^ reported that a remarkable diversity existed among olive varieties for most of traits examined in their study such as oil yield, acidity, peroxide index, and total phenolic parameters. Their results indicated that, a high diversity in the composition and quality parameters of different olive cultivars, even in a single region may be found. After the Arbequina and Arbosana cultivars, Koroneiki is considered among the most widely recognized olive cultivars around the world. For example, Koroneiki cultivar alone covers 50–60% of all olive fruits are used for production of olive oil in Greece^7,8^. Koroneiki is also cultivated in Iran and it has an important oil yield^9^.

Weather changes undoubtedly is the foremost impending environmental issue which the globe is facing with, nowadays. The variations in climate temperature can have remarkable influence on wildlife, diverse ecosystems, and food chains^10^. In specific for olive fruits, environmental changes can make significant impacts on crop yields, pest and weed ranges, and the growing season length^11^. Olive trees can blossom with a widespread range of territories and under different atmosphere conditions and frequently with absent of any watering. Several studies reported that the environmental changes have impacted the olive tree and olive oil^12,13^. Climatic condition variations (such as significant temperature changes) may affect the physiological property and behavior of the olive tree. Then, this behavioral alteration can affect the fruit ripening process, subsequently affects both the amount and the quality properties of oil existing in olives. For example, Panelli *et al*.^14^ showed that the weather conditions have an important role on the quality of olive oil mainly through modifying some constituents of aliphatic alcohols and phenols. In another study, changes of several fatty acids, sterols, alcohols, and hydrocarbons was observed by variations in the length of olive trees which was mainly due to the exposure of olive fruits to different weather conditions^15^. Also, Angerosa *et al*.^16^ reported a clear relationship between the level of some oil compounds (such as squalene, sterols, oleic acid and long-chain esters and phytol, and triacylglicerols) and weather conditions (e.g., autumn temperatures and rainfall over year).

Thus, due to the great importance of weather changes on the olive fruit properties and consequently on the extracted olive oil, in this study, three different temperatures were applied to simulate weather changes. The main goal of our work was to evaluate changes in the oil quality extracted from olives obtained from two different origins subjected to different temperatures and time periods.

## Materials and Methods

Ethanol and n-heptane were purchased from Merck (Germany). Hexane, diethyl ether, and cyclohexane were obtained from Sigma Aldrich (USA). Sodium hydroxide and potassium hydroxide were provided by Tirachem (Iran). Chloroform and acetic acid were supplied by Merck (Germany). All other chemicals were of analytical grade.

### Sample preparation

Two stocks of olive fruit (Koroneiki cultivar) were harvested at an optimum ripeness in the regions of Gorgan and Kordkuy (located in northeast of Iran). To evaluate the effects of weather temperature changes, freezing condition, and duration of keeping olives before oil extraction on the quantity and quality of extracted oil, the olives were subjected to three different temperatures (−18, 5, and 20 °C), for various time intervals (0, 20, 40, and 60 days), in a factorial design procedure. Upon experiment day, the oil extraction was performed using a pilot extraction plant, Abencor (Spain) equipped with a hammer crusher. The obtained oils were decanted, poured into dark glass bottles and kept in the refrigerator until experiments.

### Determination of olive oil quality indices

The moisture content of olive oil samples was measured according to AOAC methods 934.01^17,18^. Acidity was analyzed according to the procedure proposed by ISO660^19^. Peroxide measurement was carried out according to the method proposed by ISO3960 (2001). Total phenolic content was measured according to the procedure recommended by Zribi *et al*.^20^. To study olive oil oxidative stability, TBA value was analyzed according to the method suggested by Gomes *et al*.^21^. The color quality of olive oil obtained from different treatments was investigated using a Lovibond colorimeter with the CIELAB^22,23^.

### Statistical analysis

Data were subjected to one-way analysis of variance (ANOVA). The significant difference was assessed at 0.05 probability level and differences between treatments were tested using the Tukey’s test. The data were reported as mean values ± standard deviation (SD). Minitab (ver 16) was applied for all statistical analyses.

## Results and Discussion

### Influence of olive origin and temperature on oil yield

**Figure 1:**
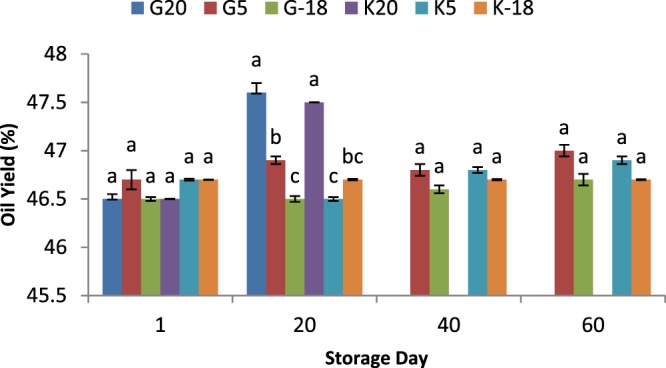
The olive oil yield from two sources and kept at different temperatures for 60 days; a,b,c means within a group which are not followed by a common superscript letters are significantly different (*P* < 0.05).

The harvesting time, weather and time period of olive storage are among important factors affecting the oil yield. Generally, olives remained in drier weather with higher temperatures would experience higher water loss until extraction process, resulting in an increase of oil yield (% of dry mass). One kg of olive fruits subjected to different treatments was randomly selected and processed to extract its oil. Upon oil production, the volume of obtained oil was measured by using a graduated tube and considering 0.915 kg/L (at ambient temperature) as the density of olive oil, the oil yield was calculated and expressed as the percentage of initial fruit weight. Our results showed a non- significant (*P* < 0.05) increase in the olive oil yield with the increase in time interval (Fig. 1). At 20^th^ day of storage, the fruits subjected to 20 °C presented significantly (*P* < 0.05) higher oil yields (47.5 and 47.6% of dry mass) compared to other samples. For other time periods also the oil yield was higher for samples remained at higher temperature (5 °C) than freezing (−18 °C) temperature, but their difference (*P* < 0.05) was not significant. In general, the weather with a higher temperature would lead to the raise in moisture release through evaporation and increase of the ratio of oil within the fruit. As well it can be mentioned that higher temperatures can result in structure degradation in some extent which facilitates the release of the oil during the extraction process^24^. In this experiment, no significant difference (*P* < 0.05) was found between the origins of olive from Gorgan and Kordkuy in terms of their oil yield.

### Influence of olive origin and temperature on oil moisture content

**Figure 2:**
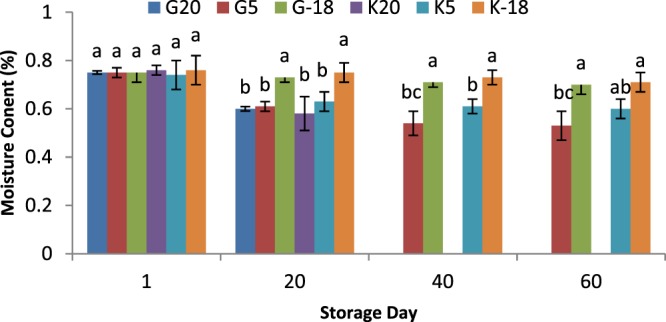
Moisture content of olive oil extracted from two sources and kept at different temperatures for 60 days; a,b,c means within a group not followed by a common superscript letters are significantly different (*P* < 0.05).

The moisture content is a significant quality parameter of olive oil regarding purity and shelf life aspects. Previous studies have shown that the presence of water plays a remarkable role in hydrolysis of fatty acids as well as oil oxidation, leading to the rancidity and off-flavor. Therefore, decreasing the moisture level of produced oil is of great importance in industry. Keeping olives at the freezing temperature (−18 °C) had no significant effect (*P* > 0.05) on the moisture content of extracted oil (compared to the oils obtained from fresh fruits), for all time intervals, while for other temperatures (5 and 20 °C), mainly a significant decrease was observed, as displayed in Fig. 2. The moisture content of olives subjected to the extraction generally has a direct influence on the moisture level of produced oil. The higher moisture content results in the more transfer of moisture to the oil during the extraction process. Accordingly, the obtained results can be explained by the fact that freezing temperature kept the moisture level in more constant status and prevented evaporation occurred at temperatures of 5 °C and more at 20 °C.

### Influence of olive origin and temperature on oil acidity

**Figure 3:**
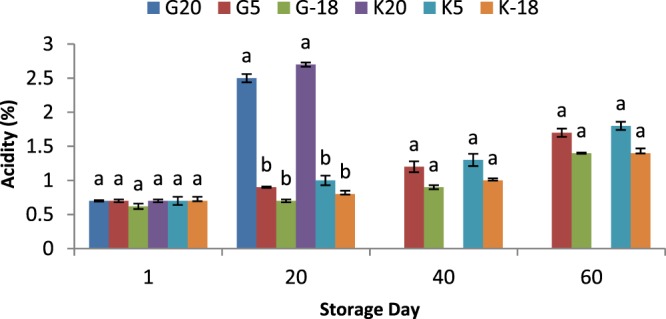
Acidity of olive oil extracted from two sources and kept at different temperatures for 60 days; a,b,c means within a group which are not followed by a common superscript letters are significantly different (*P* < 0.05). Gorgan olive at 20 °C (G20); Gorgan olive at 5 °C (G5); Gorgan olive at -18 °C (G-18); Kordkuy olive at 20 °C (K20); Kordkuy olive at 5 °C (K5); Kordkuy olive at −18 °C (K-18).

The increase of acidity level of oils which is mainly due to the activity of hydrolytic enzymes is commonly monitored to study the quality deterioration of oils^25^. In this work, a slight increase in acidity of olive oils kept at cold (5 °C) and freezing (−18 °C) temperatures at 20^th^ day and a significant raise (*P* < 0.05) for these samples at 40^th^ and 60^th^ days, compared to the fresh samples was observed (Fig. 3). The increase of acidity for oils extracted from samples kept at 20 °C was very sharper than other temperatures which well showed the remarkable influence of high temperatures on the acidity of extracted oils. For every time interval, the trend of acidity of oil samples was 20 °C > 5 °C > 18 °C. This result is in consistent with the findings of several studies conducted to study the impact of olive fruits storage conditions on extracted oil quality. Keeping of Picual olives at 5 °C preserved the level of acidity, peroxide, and ultraviolet absorbance of the extracted oil within the quality limits suggested for a 45-day-fruit storage^26^. In addition, when Yousfi *et al*.^27^ studied the effect of cold conditions (3 °C) on the composition and quality of virgin olive oil obtained from Arbequina olives. Storing fruits at 3 °C led to the highest quality by delaying the deterioration in the fruits before extraction, compared to ambient temperature. Similarly, it was shown that keeping olives at 5 °C for 30 days, maintained the initial quality of oils, while olives remained at room temperature for 15 days showed the oil quality deterioration^28^.

The raise of oil acidity during storage of olive fruit or oil itself is mostly associated with the increase in storage temperature and consequently the increase in the activity of fungal lipase^26,29^. Similarly, the findings of other studies demonstrated that the increase in oil acidity before extraction is positively related to the raise in storage temperature for diverse olive varieties^26,28^. The hydrolytic activity of microorganisms and secretion of lipolytic enzymes which can be accelerated at higher temperatures, results in the release of free fatty acids from triacylglycerol compounds; and is a major cause of increasing in acidity of oils at higher temperatures^28^. The oil samples obtained from Kordkuy cultivar showed a higher acidity level than olive oils from Gorgan cultivar, which this difference probably is due to the weather differences between these two areas. In this regard, the higher moisture content of oils extracted from Kordkuy may contribute to its higher acidity.

### Influence of olive origin and temperature on peroxide value of olive oil

**Figure 4:**
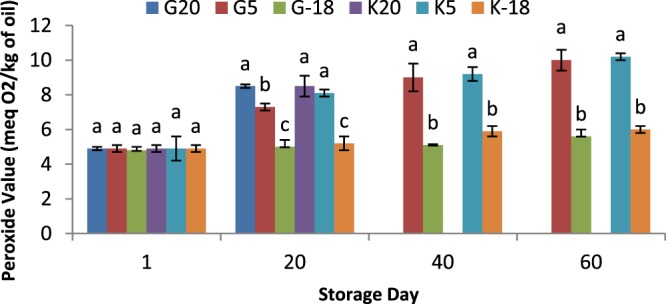
Peroxide value of olive oil extracted from two sources and kept at different temperatures for 60 days; a,b,c means within a group not followed by a common superscript letters are significantly different (*P* < 0.05).

Peroxide value of oils extracted from stored olives raised during storage time, compared to olive oil obtained from fresh olives (Fig. 4). Like the results of acidity evaluation, the peroxide value trend was probably a result of the higher activity of lipoxygenase enzymes at higher temperatures. At 20^th^ day of storing olives at 20 °C, the peroxide value for olive oil was the highest (8.5 meq O_2_/kg of oil) compared to other treatments at this day which well showed the effect of higher temperatures on peroxide value. For results obtained at 40^th^ and 60^th^ days, the higher values of peroxides for extracted olive oils were found at 5 °C rather than −18 °C. Thus, regarding peroxide value, a cold weather (below −10 °C) may not be detrimental for olive fruits in terms of the quality of extracted oil. Our results were in consistent with the study of Garcia *et al*.^26^ which showed a trend of increasing in the peroxide values for the oils extracted from olives kept at different temperature (ambient temperature >8 °C > 5 °C) for the majority of experiment’s period (60 days). Similarly, the oils extracted from olives remained at various temperatures (0, 5, 7.5 °C) showed an increase in the peroxide value during 60 days of storage at higher temperatures^30^. However, the raise in peroxide level was significant (*P* < 0.05) only for olives subjected to 7.5 °C, compared to the oil extracted from fresh olives.

The oil samples obtained from Gorgan cultivar showed a slightly lower peroxide value compared to Kordkuy cultivar samples subjected to different temperatures which was consistent with the results for acidity. This could be due to the higher moisture content of Kordkuy olive oils, leading to a higher hydrolyzed triglycerides content, resulted in a higher sensitivity to autoxidation and subsequently the raise in peroxide value. In the current study, the peroxide value did not exceed the limits set by the regulation with a maximum of 30.6 meq O_2_/kg of oil for all treatments studied^31^.

### Influence of olive origin and temperature on phenolics content

**Figure 5:**
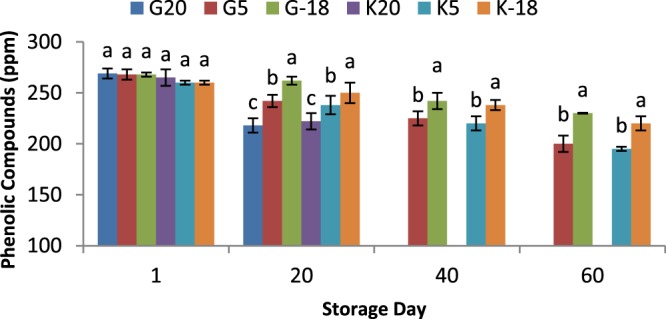
Total phenolics of olive oil extracted from two sources and kept at different temperatures for 60 days; a,b,c means within a group not followed by a common superscript letters are significantly different (*P* < 0.05).

Phenolic compounds in olive oil composition are vital for protecting it against oxidation^32,33^. They perform this protective activity through exposing themselves to the oxidation agents through diverse mechanisms relied on transfer of hydrogen atom, radical scavenging, and metal-chelating^34^. Hydroxytyrosol and its derivatives are the key phenolic compounds with such performance and their content in olive oil should be at least 5 mg/20 g oil^35^. The total phenolic content decreased significantly (*P* < 0.05) as the olive fruit storage time progressed (Fig. 5). In addition, the influence of temperature on phenolics content was 20 °C > 5 °C > −18 °C. Variations in the phenolics level, during storage of olive fruit is the consequence of oxidative and hydrolytic activities. Accordingly, it was explained that decrease of temperature and oxygen availability are major parameters which should be considered to reduce the phenolic compounds loss^36^. Therefore, it can be hypothesized that cooler weather with a lower wind velocity can better protect the phenolic compounds of subjected olives. In a study, the phenolic compounds in the olive oils decreased as the olive storage period increased. The highest negative effect of keeping olives at 20 °C (90.31%, 95.78% and 91.73%) compared to the samples subjected to 4 °C (22.5%, 25.93% and 26.17%) well showed the significant effect of temperature on reduction of phenolic compounds^36^.

Several endogenous enzymes (such as β-glucosidases, peroxidases, lipases, pectinases, and lipoxygenases) which are mainly liberated from disrupted fruit tissues because of mechanical damages during harvest or provided by pathogenic micro-organism during storage are very important. These enzymes are the main cause of metabolisms related to the decrease in phenolic compounds^36^. Among which two classes of endogenous enzymes including β-glucosidase hydrolysing phenolic glycosides and oxidoreductase enzymes are the most important enzymes in degradation of phenolic compounds. It should be noted that phenolic compounds are very important for improving the stability and inhibition of autoxidation which even contribute into the sensory properties of oils^37^. The warmer weather can remarkably increase the activity of phenolic deteriorating enzymes and affect the produced oil quality. Increasing the time of olive storage until oil extraction would also give more opportunity to these deteriorating enzymes to degrade phenolic compounds. The measurement of total phenolic content showed no significant difference (*P* < 0.05) between olives with different origin (Gorgan and Kordkuy) for every time interval and temperature. Similarly, for both cultivars, in every storage period, the level of phenolic compounds for extracted oil samples was as: −18 °C > 5 °C > 20 °C.

### Influence of olive origin and temperature on thiobarbitoric acid (TBA) values of extracted oils

**Figure 6:**
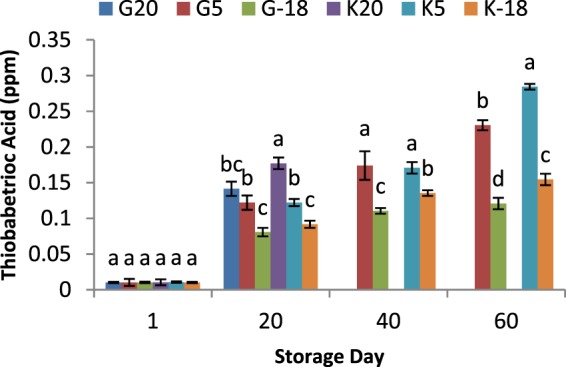
Thiobarbitoric acid (TBA) values of olive oil extracted from two sources and kept at different temperatures for 60 days; a,b,c means within a group which are not followed by a common superscript letters are significantly different (*P* < 0.05). Gorgan olive at 20 °C (G20); Gorgan olive at 5 °C (G5); Gorgan olive at −18 °C (G-18); Kordkuy olive at 20 °C (K20); Kordkuy olive at 5 °C (K5); Kordkuy olive at −18 °C (K-18).

TBA value is used to assess the extent of secondary oxidation substances in oil and oily foods^21,38^. The degradation of hydroperoxides which produces secondary oxidation compounds as well as transformation of primary lipid oxidation products to secondary lipid oxidation substances during the storage of oily products leads to an increase in the level of secondary oxidation products^39–41^. The TBA value rose for all olive oil samples as the time of storage elapsed (Fig. 6). However, the TBA levels were well below the rancidity onset which usually occurs at TBA levels of 1.00 and higher, indicating the stability of olive oil during time periods studied. According to Table 1, after every period of storage, the higher temperatures led to the higher TBA values for extracted oils and the TBA value for any storage period was 20 °C > 5 °C > −18 °C. Furthermore, TBA assay showed a significant difference (*P* < 0.05) between TBA values of oils extracted from Gorgan and Kordkuy cultivars. Like the results of acidity and peroxide value, the olives from Gorgan cultivar showed a more potential to keep their quality against weather temperature fluctuations, than Kordkuy cultivar in terms of TBA value.

**Table 1:** Development of rancidity (TBA value as malonaldehyde mg/kg sample) of oil extracted from olives of two sources and kept at different temperatures for 60 days.

Sample Codes	Days			
	1	20	40	60
G20	0.0102^a^	0.1414^b^	ND	ND
G5	0.0103^a^	0.1221^c^	0.1740^a^	0.2305^b^
G-18	0.0103^a^	0.0808^d^	0.1105^c^	0.1208^d^
K20	0.0105^a^	0.1771^a^	ND	ND
K5	0.0107^a^	0.1221^c^	0.1708^a^	0.2844^a^
K-18	0.0102^a^	0.0918^d^	0.1355^b^	0.1545^c^

ND: Not Defined. a,b Means within a column which are not followed by a common superscript letters are significantly different (*P* < 0.05).

### Influence of olive cultivar and temperature on oil color

Chlorophylls are known as the main cause of the greenish color of specific olive oils and carotenes are the principal cause of its yellow coloration. These pigments also have a substantial effects on the oxidative activity of oils constituents because of being antioxidant in the dark and prooxidant in the light^31^. In addition, these pigments greatly contribute in other sensory properties of olive oil. The results (Table 2) showed that with the increase of olive storage time, the values of “L” index slightly increased indicating the rise in oil brightness which could be due to the autoxidation and subsequently the degradation of chlorophylls. Since the most of enzymatic and chemical activities are stopped at −18 °C, the lowest changes in “L” value were found for this temperature, compared to 20 °C and 5 °C. The oils obtained from Gorgan and Kordkuy cultivar showed similar behaviors for the “L” value changes and no significant difference (*P* < 0.05) was found.

**Table 2:** Color parameters of olive oil from two sources and kept at three different temperatures for 60 days.

Index	Sample Codes	Days			
		1	20	40	60
L*	G20	20.00^a^	20.40^a^	ND	ND
	G5	20.00^a^	20.35^a^	20.88^a^	21.78^a^
	G-18	20.05^a^	20.18^a^	20.38^b^	20.65^b^
	K20	20.05^a^	20.45^a^	ND	ND
	K5	20.05^a^	20.38^a^	21.10^a^	21.80^a^
	K-18	20.00^a^	20.18^a^	20.38^b^	20.70^b^
a*	G20	8.03^a^	8.07^a^	ND	ND
	G5	8.02^a^	8.05^a^	8.07^a^	8.12^a^
	G-18	8.02^a^	8.04^a^	8.05^a^	8.06^b^
	K20	8.05^a^	8.09^a^	ND	ND
	K5	8.05^a^	8.07^a^	8.07^a^	8.17^a^
	K-18	8.06^a^	8.06^a^	8.07^a^	8.14^a^
b*	G20	1.13^a^	1.02^a^	ND	ND
	G5	1.13^a^	1.03^a^	0.92^a^	0.82^b^
	G-18	1.12^a^	1.05^a^	1.02^a^	0.96^a^
	K20	1.12^a^	1.01^a^	ND	ND
	K5	1.12^a^	0.99^a^	0.91^a^	0.75^b^
	K-18	1.09^a^	1.03^a^	0.98^a^	0.92^a^

ND: Not Defined. a,b Means within a column which are not followed by a common superscript letters are significantly different (P < 0.05).

Gorgan olive at 20 °C (G20); Gorgan olive at 5 °C (G5); Gorgan olive at -18 °C (G-18); Kordkuy olive at 20 °C (K20); Kordkuy olive at 5 °C (K5); Kordkuy olive at −18 °C (K-18).

The “a” value of olive oils for all temperatures increased at higher storage times indicating the raise in the redness and decrease in greenness of oil samples. This could be due to degradation of chlorophylls during storage of olives. The source of olives could make a difference in “a” induce after 60 days of storage and Kordkuy cultivar olive remained at 5 °C presented the lowest “a” value (8.06). A decrease in “b” value was found as storage of olives proceeded indicating the decrease in yellowness of oil samples (Table 2). The higher temperatures could intensify the decrease of “b” value due to higher autoxidation of carotenoids compared to lower temperatures. Kordkuy cultivar olive presented a slightly lower “b” value for all storage conditions compared to Gorgan cultivar samples. According to the results of color experiment, increase of storage time decreased the color quality of oil samples, particularly at higher temperatures.

## Conclusion

Weather conditions and temperature fluctuations have substantial effects on the quality of harvested olives and their corresponding oils. This study showed that while no negative effect of storage of olive fruits at −18 °C temperature was observed on the extracted oil, the storage of olive samples at higher temperatures (5 °C, 20 °C) significantly (*P* < 0.05) increased the acidity, peroxide value, and TBA value of the extracted olive oil. The trend of oils with higher quality considering all parameters studied was −18 °C > 5 °C > 20 °C. Also, as the storage time of olive fruits before extraction increased, the quality of obtained oil was decreased. Therefore, shortening the storage time of fruits olive as low as possible, would prevent the negative effects of temperature fluctuations, especially for areas with warmer weather on the quality of oils. Different olive cultivars can significantly affect the behavior of olive fruit and its oil in storage conditions mainly due to the differences in the composition of oils.
